# mTORC1 controls long-term memory retrieval

**DOI:** 10.1038/s41598-018-27053-5

**Published:** 2018-06-08

**Authors:** Magdalena Pereyra, Cynthia Katche, Ana Belén de Landeta, Jorge H. Medina

**Affiliations:** 1 Instituto de Biología Celular y Neurociencias, Facultad de Medicina, UBA, Paraguay 2155, 3 piso, Buenos Aires, C1121ABG Argentina; 2 Departamento de Fisiología, Facultad de Medicina, UBA, Paraguay 2155, 7 piso, Buenos Aires, C1121ABG Argentina

## Abstract

Understanding how stored information emerges is a main question in the neurobiology of memory that is now increasingly gaining attention. However, molecular events underlying this memory stage, including involvement of protein synthesis, are not well defined. Mammalian target of rapamycin complex 1 (mTORC1), a central regulator of protein synthesis, has been implicated in synaptic plasticity and is required for memory formation. Using inhibitory avoidance (IA), we evaluated the role of mTORC1 in memory retrieval. Infusion of a selective mTORC1 inhibitor, rapamycin, into the dorsal hippocampus 15 or 40 min but not 3 h before testing at 24 h reversibly disrupted memory expression even in animals that had already expressed IA memory. Emetine, a general protein synthesis inhibitor, provoked a similar impairment. mTORC1 inhibition did not interfere with short-term memory retrieval. When infused before test at 7 or 14 but not at 28 days after training, rapamycin impaired memory expression. mTORC1 blockade in retrosplenial cortex, another structure required for IA memory, also impaired memory retention. In addition, pretest intrahippocampal rapamycin infusion impaired object location memory retrieval. Our results support the idea that ongoing protein synthesis mediated by activation of mTORC1 pathway is necessary for long but not for short term memory.

## Introduction

The ability to recall past events is a major determinant of survival strategies in all species. Memory retrieval is the act of making stored information available for immediate use and is considered to be a complex and probably multistage process by which previously acquired information is reactivated from a latent state to a state that permits its expression. Not always but in most cases it is also accompanied by a behavioral output in the animal that constitutes the experimental assessment of a given memory. Memories reactivation at the time of retrieval seems to be a rapid process since animals may retrieve as soon as they receive, usually without notice, the CS or other cues including US remindful stimuli, another related memory recall or an emotional context associated.

A body of evidence suggest that long-term memories (LTM) rely on functional and structural changes at synapses, as Ramón y Cajal had proposed more than a century ago^1–3^. Neurons and their synapses that are reactivated when the animals are demanded to retrieve are believed to be those that have been changed through the molecular processes that underlie memory formation^1,4^. But in contrast to this initial stage, the information about the molecular mechanisms of memory retrieval is surprisingly scarce and fragmentary, despite its necessity for utilizing learned information. Some neurotransmitters and their receptors including metabotropic and AMPA glutamate receptors are required for memory retrieval in the hippocampus and related structures, while β-adrenoreceptors and cholinergic muscarinic receptors have neuromodulatory action^5–11^. In addition, several protein kinases including ERK1/2, PI3K and PKA are necessary for retrieval of fear-motivated memories in the hippocampus^9,10,12^. Despite the fact that protein synthesis has been extensively reported as a key factor in memory consolidation and reconsolidation, its role on memory retrieval remains controversial. Experiments using anisomycin as a general protein synthesis inhibitor (PSI) yielded negative or conflicting results^13–15^. However, Nader and coworkers^11^ recently found that pretest intramygdala infusion of anisomycin or rapamycin (rapa), a specific mTORC1 inhibitor, impaired auditory fear memory retrieval. These findings suggest that ongoing protein synthesis induced by mTORC1 pathway is required in the amygdala to enable memory retrieval.

mTORC1 is a serine/threonine kinase complex that modulates cell proliferation and growth, metabolism, autophagy, and mRNA translation initiation^16^. In the brain it has a prime function in regulating synaptic plasticity and memory formation, via the control of protein synthesis, including dendritic translation of synaptic proteins^17–20^.

Therefore, to further investigate the role of mTORC1 in memory retrieval we determined whether mTORC1 is required for retrieval of an inhibitory avoidance (IA) task, a fear-motivated and hippocampus-dependent learning experience. We found that inhibition of hippocampal mTORC1 using infusions of rapa before test (TS) sessions at 1, 7 or 14 days after training provoked a marked and reversible impairment of recent and remote IA LTM expression, but not when mTORC1 inhibition was performed before testing for short-term or for 28 days long-term memory. Inhibition of mTORC1 before LTM testing in the retrosplenial cortex, another brain region involved in IA LTM retrieval^21^, also resulted in transient impairment in memory expression. LTM expression of object location (OL) was also affected by pretest inhibition of mTORC1.

## Results

### Pretest infusion of rapamycin or emetine impaired IA LTM retrieval

We chose to use IA training because it is acquired in a single and brief training session, which makes it ideal for investigating the neural and molecular events associated with memory retrieval without interference from previous expression of the learned behavior which occurs in multi-trial tasks^22^.

**Figure 1:**
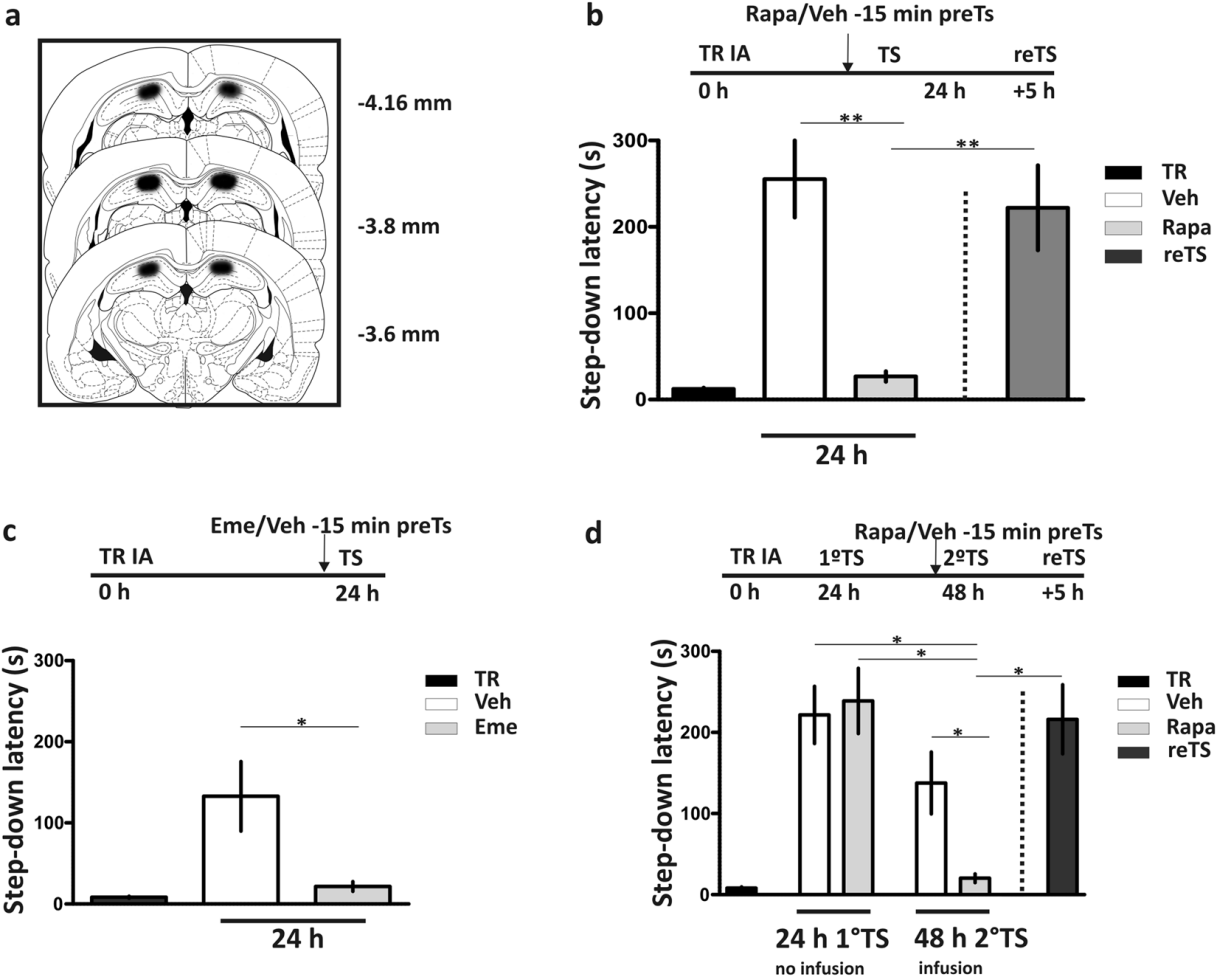
Protein synthesis is required for inhibitory avoidance long term memory retrieval. (**a**) Schematic representation of rat brain section at three rostrocaudal planes (AP: −3.6; −3.8 and −4.16 mm from Bregma) showing the extent of area reached by the infusion in the dorsal hippocampus (CA1). The drawings were adapted from Paxinos and Watson^48^. (**b**) Intrahippocampal rapamycin (Rapa) infusion 15 min before test (TS) session carried out 24 h after training (TR) impairs inhibitory avoidance long term memory (IA-LTM) retrieval. **p < 0.01, vehicle (Veh) vs. Rapa group and Rapa vs. reTS, one-way ANOVA followed by Newman-Keuls Comparison Test, n = 5-6. Data are expressed as mean ± SEM of TR or TS session step-down latency. (**c**) Intrahippocampal infusion of emetine (Eme) 15 min before TS also affects 24 h IA-LTM retrieval. *p < 0.05, Veh vs. Eme group, unpaired Student’s t Test, n = 6-7. Data are expressed as mean ± SEM of TR or TS session step-down latency. (**d**) Reversible blockade of an already expressed IA-LTM memory by intrahippocampal Rapa infusion 15 min before TS session 24 h after the first TS without any infusion. **p < 0.01, Veh vs. Rapa, 1°TS Veh vs. Rapa, and Rapa vs. reTS groups; ***p < 0.001, Rapa vs. 1°TS Rapa groups, one-way ANOVA followed by Newman-Keuls Test, n = 9-10. Data are expressed as mean ± SEM of TR or TS session step-down latency.

Confirming and extending recent findings in auditory fear memory retrieval using intraamygdala infusions of PSIs^11^, we found that intrahippocampal infusions (Fig. 1a) of two different types of PSI, emetine (eme) or rapa, 15 min before an IA TS session performed 24 h post training greatly impaired the expression of IA LTM (Fig. 1b, p < 0.01, rapa compared to vehicle (veh) rats, Newman-Keuls Comparison Test after one-way ANOVA, n = 5–6 and Fig. 1c, p < 0.05, eme compared to veh rats, Student’s t Test, n = 7–8). Similar impairments in memory retrieval were obtained with the infusion of the specific inhibitor of mTORC1, rapa, or the administration of eme, which is a general PSI. The deficit on memory retrieval caused by rapa was transient, because a subsequent TS session performed 5 h after the first one showed normal IA retention performance (Fig. 1b, dark gray bar). Moreover, this reversible blockade of memory retrieval by intrahippocampal rapa was also seen in trained rats that expressed IA memory at a 24 h TS and the inhibitor of mTORC1 was given 15 min before a second TS carried out 48 h after training (Fig. 1d, p < 0.05, rapa vs. veh groups, one-way ANOVA followed by Newman-Keuls Comparison Test, n = 9–10). When infused outside the intended area in the dorsal hippocampus rapa did not affect retrieval. For instance, it did not impair retention performance when infused in the dorsal thalamic region (n = 3) or the hippocampal hilus region (n = 2) (data not shown). Taking together, these findings indicate that ongoing protein synthesis is required in the dorsal hippocampus to retrieve IA memory.

**Figure 2:**
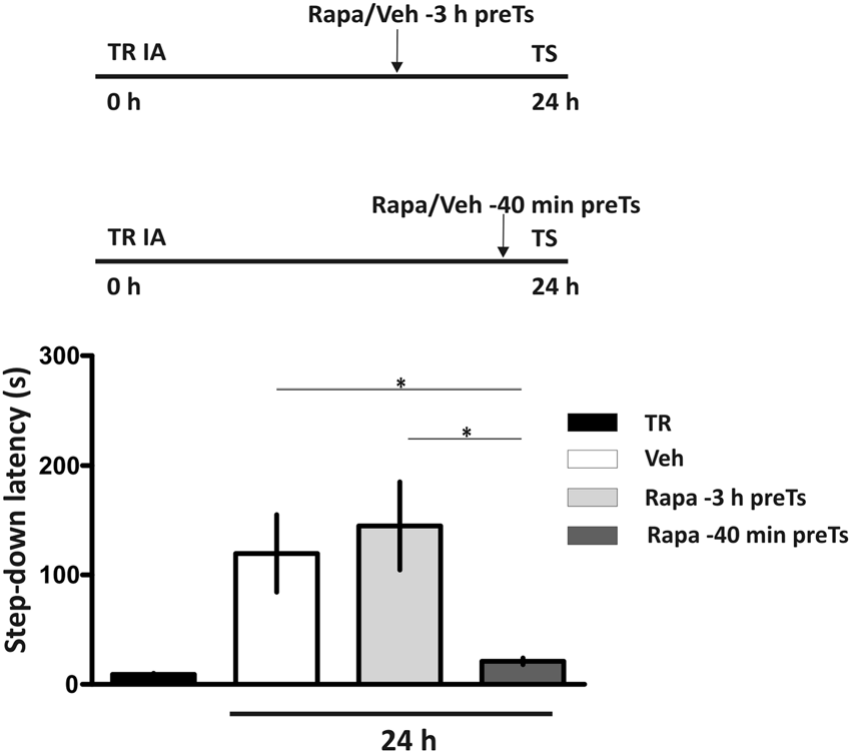
Dynamics of rapamycin effect. Rats were bilaterally infused with rapamycin (Rapa) or saline (Veh) into the hippocampus 3 h or 40 min before a test (TS) session carried out 24 h after training (TR). *p < 0.05, Veh vs. Rapa 40 min and Rapa −3 h vs. Rapa-40min groups; p > 0.05, Veh vs. Rapa groups, one-way ANOVA followed by Newman-Keuls Comparison Test, n = 9 per group. Data are expressed as mean ± SEM of TR or TS session step-down latency.

The effect of intrahippocampal rapa on IA LTM expression was also seen when the compound was infused 40 min before subjecting the animals to a TS session carried out 24 h after training (Fig. 2, p < 0.05, rapa 40 min compared to veh group, Newman-Keuls Comparison Test after one-way ANOVA, n = 9 per group). However, administration of rapa into the dorsal hippocampus 3 h before the TS session provoked no impairment on memory retrieval (Fig. 2, p > 0.05, rapa3h vs. veh animals, Newman-Keuls Comparison Test after one-way ANOVA, n = 9 per group).

### Rapamycin affected LTM, but not STM recall

**Figure 3:**
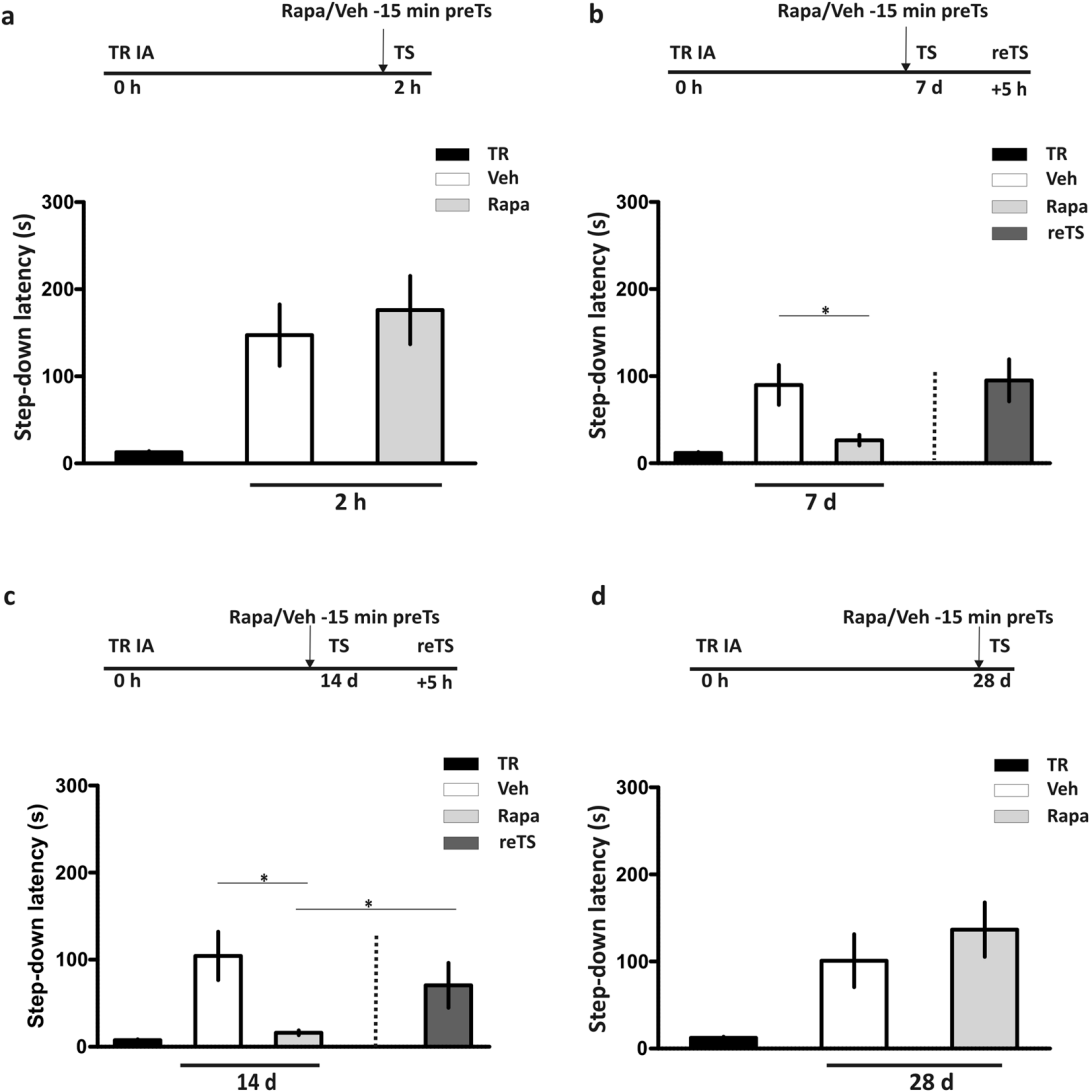
Rapamycin effect depends on the age of the memory retrieved. (**a**) Rapamycin (Rapa) was administered 15 min before a test (TS) session carried out 2 h after training (TR). p > 0.05, Vehicle (Veh) vs. Rapa short term memory groups, unpaired Student’s t Test, n = 8–12. Data are expressed as mean ± SEM of TR or TS session step-down latency. In the next experiments, Rapa was administered 15 min before a TS session carried out (**b**) 7 (*p < 0.05, Veh vs. Rapa 7d and Veh vs. reTS groups, one-way ANOVA followed by Newman-Keuls Comparison Test, n = 11-12), (**c)** 14 (*p < 0.05, Veh vs. Rapa 14d groups, one-way ANOVA followed by Newman-Keuls Comparison Test,n = 6-7) or, (**d**) 28 days after TR (*p > 0.05, Vehvs. Rapa 28 d groups, unpaired Student´s t test n = 9 per group). Data are expressed as mean ± SEM of TR or TS session step-down latency.

We next determined whether the effect of rapa on memory expression depends on the age of LTM to be retrieved. As shown in Fig. 3b,c the inhibition of mTORC1 15 min before TS sessions at 7 or 14 days after training impaired IA LTM retention performance (Fig. 3b,c, p < 0.05, rapa 7d vs. veh groups and rapa 14d vs. veh groups, Newman-Keuls Comparison Test after one-way ANOVA, n = 11-12 and n = 6-7 respectively). In both cases, a retest carried out 5 h after TS restored retrieval (Fig. 3b,c, dark gray bars). At 28 days after IA training, some authors had reported that memory expression becomes muscimol and CNQX-insensitive after pretest intrahippocampal infusions^23,24^, suggesting that IA memory might become hippocampus-independent. Consistent with these previous works, the intrahippocampal infusion of rapa 15 min before 28 days TS provoked no alterations in memory retrieval (Fig. 3d, p > 0.05, rapa 28d vs. veh groups, Student’s t Test, n = 9 per group). Moreover, mTORC1 is not required for retrieval of short-term memory (STM), because the infusion of rapa into the dorsal hippocampus 15 min before a TS session performed 2 h after training did not alter IA memory retention (Fig. 3a, p > 0.05, Student’s t Test, n = 8–12).

**Figure 4:**
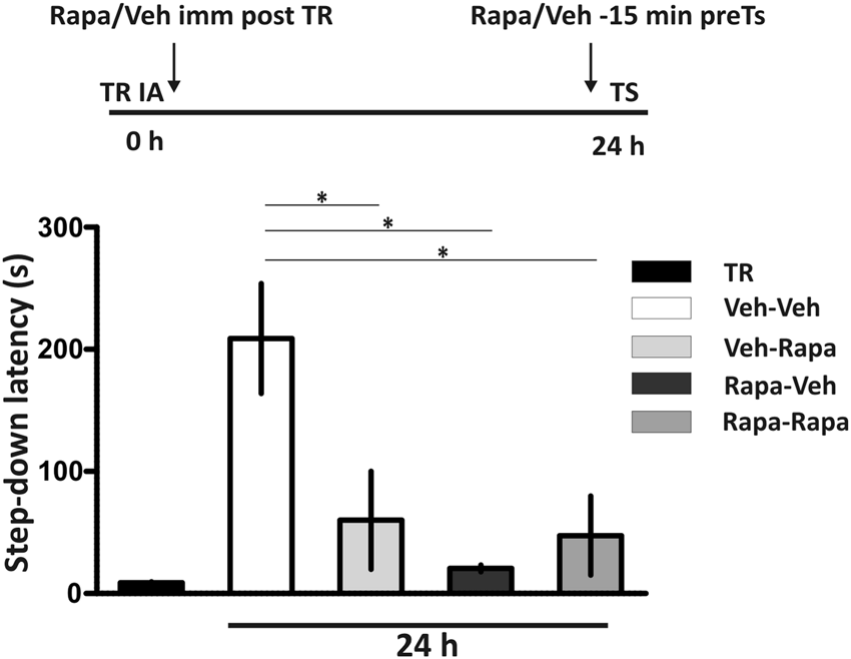
Intrahippocampal infusion of rapamycin 15 minutes before test does not alter neuronal internal state of the animals. Rats were bilaterally infused with rapamycin (Rapa) or saline (Veh) into hippocampus CA1 at two different time points: immediately after training (TR) and 15 min before a test (TS) session carried out 24 h after the TR. **p < 0.01, for Veh-Veh vs. Veh-Rapa, Veh-Veh vs. Rapa-Veh and Veh-Veh vs. Rapa-Rapa groups, one-way ANOVA followed by Newman-Keuls Comparison Test; n = 7-8. Data are expressed as mean ± SEM of TR or TS session step-down latency at 24 h after TR.

Memories have been proposed to rely on endogenous and exogenous substances state-dependent process^25,26^. The aim of the next experiment was to determine whether the impairing effect of pretest rapa on memory retrieval could be attributable to a mismatch between the pharmacological state of the animal in which information was acquired and the one in which it was recalled rather than a consequence of retrieval molecular process disruption^27,28^. Four groups of rats received two infusions of vehicle or rapa, one delivered immediately after training and the other 15 min before testing (veh-veh, veh-rapa, rapa-veh and rapa-rapa). A one-way ANOVA showed a significant effect of treatment (p < 0.01, F = 6.54). The main finding of this experiment is that rapa impaired memory when given after training or before testing as well as when delivered at both time points (Fig. 4, veh-veh differs from all the others groups, Newman-Keuls Comparison Test after one-way ANOVA), indicating that alterations in the internal state of the rat is not responsible for the effect of pretest mTORC1 inhibition on memory retrieval.

**Figure 5:**
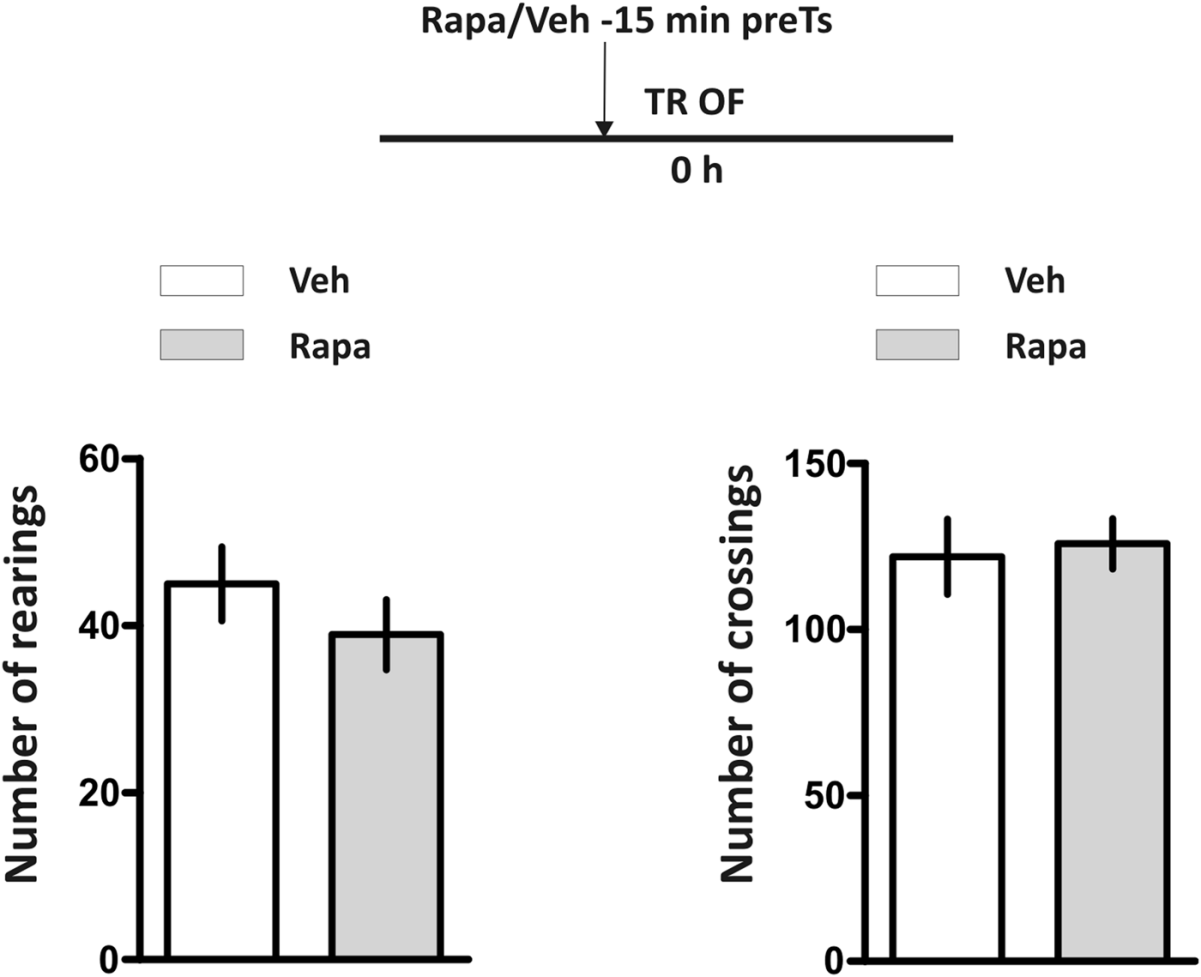
Intrahippocampal infusion of rapamycin 15 minutes before test does not affect exploratory behavior. Number of rearings (left panel) and crossings (right panel) of animals that had received intrahippocampal infusion of rapamycin (Rapa) or vehicle (Veh) 15 min before a 5 min open field session. p > 0.05, Veh vs. Rapa groups, unpaired Student´s t test, n = 12 per group. Data are presented as mean ± SEM number of crossings or rearings.

Several non-specific factors affecting sensory-motor and emotional processes can influence retention performance of an IA task. For this reason, we tested whether bilateral rapa infusion into the dorsal hippocampus affects locomotor or exploratory activity. As shown in Fig. 5, rapa had no discernible effects on these parameters in an open field (p > 0.05, Student’s t Test for both rearings and crossings of veh vs. rapa groups, n = 12 per group). Taking together with the experiment showing that pretest rapa did not affect STM retrieval (Fig. 3a), these results indicate that the impairing effect of rapa on LTM retention is not due to non-specific actions on stepping-down latency.

### Pretest rapamycin infusion on other brain regions also impaired memory retrieval

**Figure 6:**
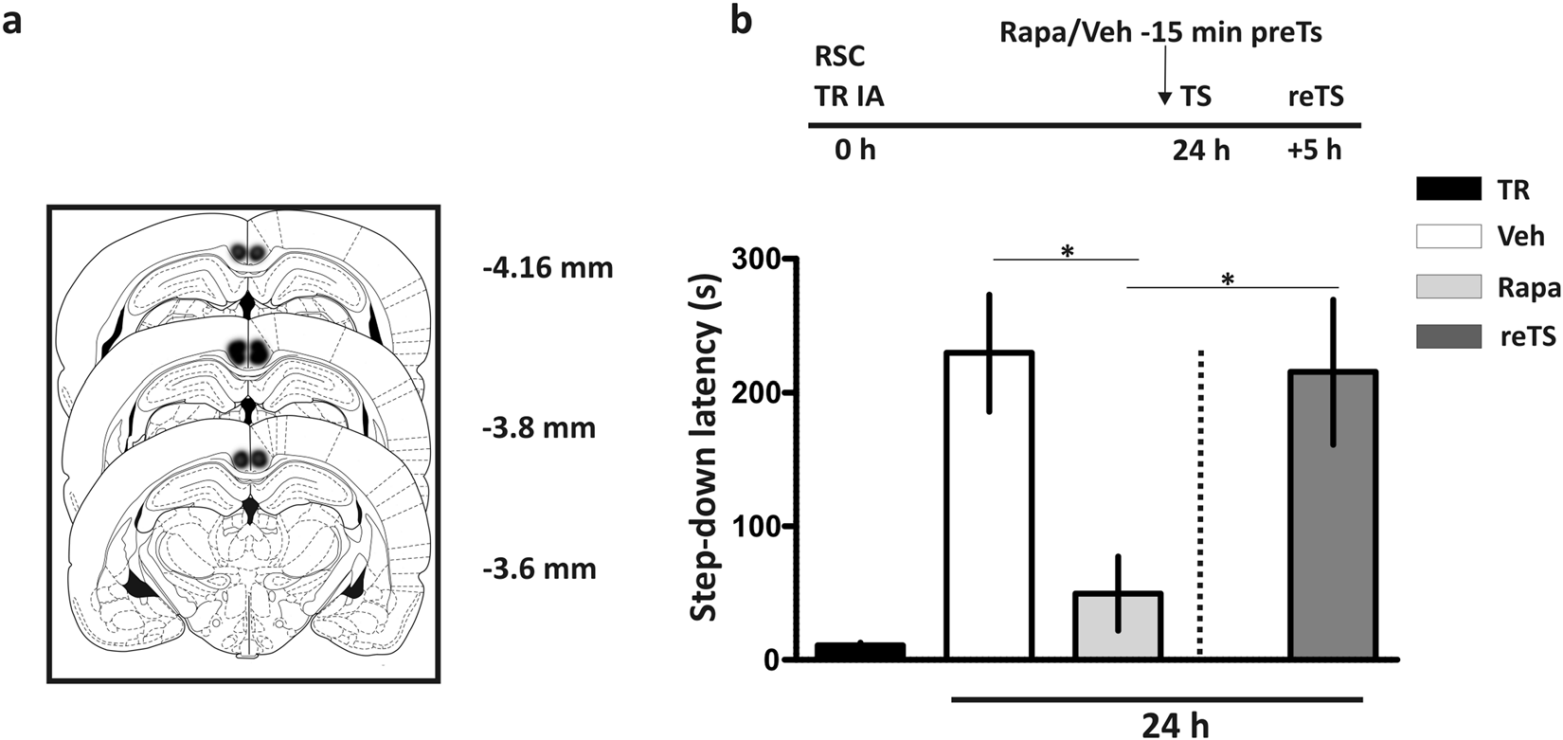
Retrosplenial cortex mTORC1 inactivation 15 minutes before test reversibly disrupts inhibitory avoidance long term memory retrieval. (**a**) Schematic representation of rat brain section at three rostrocaudal planes (AP: −3.6; −3.8 and −4.16 mm from Bregma) showing the extent of area reached by the infusion in the anterior retrosplenial cortex (RSC). The drawings were adapted from Paxinos and Watson^48^. (**b**) Rats were bilaterally infused with rapamycin (Rapa) or saline (Veh) into the RSC 15 min before a test (TS) session carried out 24 h after the training (TR). *p < 0.05, Veh vs. Rapa and reTS vs. Rapa groups; one-way ANOVA followed by Newman-Keuls Comparison Test, n = 5 per group. Data are expressed as mean ± SEM of TR or TS session step-down latency.

Several brain regions are simultaneously involved in memory retrieval, inasmuch as the memories on which retrieval is based certainly are not confined to any single brain region^29,30^. Taking this into account, we next determined whether mTORC1 in the retrosplenial cortex (RSC), a region involved in consolidation and expression of IA training^21^, is required for memory retrieval. As shown in Fig. 6b, the pretest infusion of rapa into the anterior part of RSC (Fig. 6a) transiently blocked IA memory retrieval in a reversible way (Fig. 6b, p < 0.05, Newman-Keuls Comparison Test after one-way ANOVA).

### Pretest rapamycin infusion impaired OL memory retrieval

**Figure 7:**
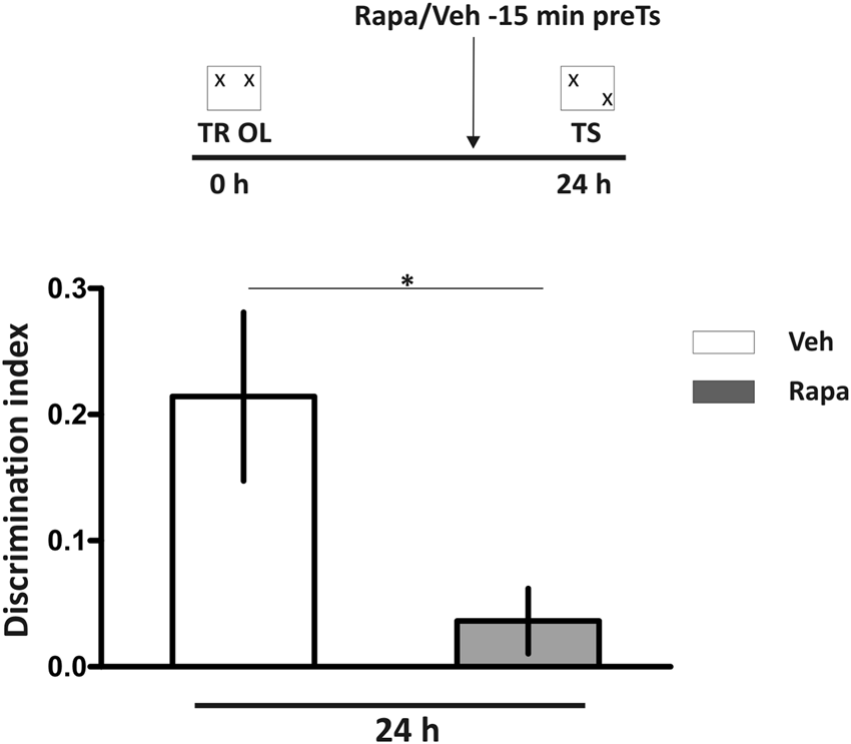
Rapamycin delivered into the hippocampus 30 minutes before test session disrupts object location long term memory retrieval. Rats were bilaterally infused with rapamycin (Rapa) or saline (Veh) into the hippocampus 30 min before a test session carried out 24 h after the training in object location task. *p < 0.05, Veh vs. Rapa groups, unpaired Student’s t Test, n = 7-8. Data are expressed as mean ± SEM of the discrimination index.

OL memory is the ability to discriminate the familiar location of previously encountered objects^31,32^. We chose this OL memory in order to assess if the disruptive effect of rapa on IA memory can be replicated in anon aversive hippocampus-dependent task. Figure 7 shows that the discrimination index significantly decreased with intrahippocampal administration of rapa 30 min before TS session of the OL task (p < 0.05, veh vs. rapa group, Student’s t test, n = 7-8).

## Discussion

The ability to recall past event is of great importance in determining our uniqueness as individuals. In this regard, retrieval is critical for memory: without its retrieval it is not possible to ensure we have a given memory. The main finding of the present study is that a general PSI, eme, and a selective inhibitor of mTORC1, rapa, infused into the dorsal hippocampus before IA testing greatly impaired memory retrieval. Given that rapa has less nonspecific effects than a general PSI like eme, we chose to use the specific inhibitor of mTORC1 to further characterize the involvement of ongoing protein synthesis on memory retrieval processing. The amnesic effect of rapa is limited to LTM, and does not depend on a drug-induced change in the internal state of the animal. Interestingly, STM retrieval does not require mTORC1 activity. This result cannot be explained by alterations in locomotor or exploratory activity, since rapa had no discernible effect on locomotion and exploration in an open field. Also, intrahippocampal rapa administration impaired memory expression of a non-aversive OL memory. Together, these findings endorse the idea that regulation of translation by mTORC1 at the moment of retrieval is required for the expression of LTM.

Remarkably, at each time point tested, the effect of rapamycin infusion on memory retrieval was transient since a 5 h re-test showed full memory recovery. This finding supports the idea that the infusion of rapa into the dorsal hippocampus produced no functional or anatomical damage and, is consistent with the fact that rapa administration 3 h prior testing had no effect on memory recall, suggesting that rapa has a limited action after its *in vivo* infusion.

Our present findings extend those of Lopez *et al*.^11^ who demonstrated that anisomycin and rapa delivered into the amygdala before testing impaired the retrieval of an auditory fear-conditioning. Because the inhibition of mTORC1 in the RSC before testing also impaired IA memory retrieval (Fig. 6) and that rapa also affect OL memory retrieval (Fig. 7), we are tempted to suggest that the requirement of mTORC1 in memory retrieval is a general feature of memory recall in mammals.

In contrast to the amnesic effect of rapa when administered 15 min pretest at 1, 2, 7 or 14 days, mTORC1 in IA memory retrieval was not required when animals were tested 28 days after training, probably because IA memory trace is no longer in the dorsal hippocampus 4 weeks after IA training. Interestingly, this time point coincides with the period of time at which the hippocampus becomes totally insensitive to the impairing effect of pretest infusions of muscimol^33^ or CNQX^23^, an AMPA/Kainate glutamate receptor antagonist. Our present findings using rapa are also consistent with experiments showing that disruption of hippocampal function mostly hindered recent LTM without affecting remote LTM^29,34,35^ although other authors had reversibly abolished remote memory recall by a brief optogenetic inhibition of CA1^36^.

STM recall is also not affected by pretest infusions of rapa into the dorsal hippocampus, suggesting that mTORC1 activity is not necessary for the retrieval of IA memory at short times after training. This finding parallels those showing that STM does not depend on new protein synthesis^3,37^. It is interesting to note here that several behavioral experiments in rodents indicate the requirement of mTORC1 in the formation, consolidation and reconsolidation of LTM, but not STM^19,22,38,39^.

Also, we have shown that the rapa effect can be extended to an OL memory task, since intrahippocampal infusion of rapa before TS session hindered OL memory retrieval as shown in Fig. 7. Considering our present findings, the effect of mTORC1 blockade on memory retrieval appears to be valence-independent and the role of mTORC1 pathway on this memory phase can be generalized to several forms of associative learning tasks.

What are the upstream regulators of the activity of mTORC1? Extracellular activators of the mTORC1 pathway with relevance to memory retrieval include brain-derived neurotrophic factor (BDNF) and glutamate^17,40,41^. Dendritic mTORC1 is activated by glutamate action on NMDA receptors to control long term potentiation (LTP)^42^ and metabotropic glutamate receptors activation results in enhancing ERK and AKT-mTOR pathways controlling protein synthesis and long-term depression^43,44^. Intracellular activators of mTORC1 such as ERK 1/2 and AKT have also important modulatory effects on retrieval of IA memory^9^.

A prevailing view tells us that retrieval involves the reconstruction of patterns of brain activity produced during initial learning. In other words, reactivation of distributed ensembles of neurons in different brain regions that were active during initial learning is necessary for the subsequent retrieval of the memory. If this is the case, how are the patterns of activity in the hippocampus generated and controlled during retrieval? Many years ago, it was suggested that all or part of the mechanisms occurring during encoding have to be reestablished at the moment of recall in order to successfully retrieve memory^27,28^. However, our findings using posttraining and/or pretest administration of rapa (Fig. 4) did not support this idea. Later on, molecular pharmacological data showed that the biochemical changes underlying IA consolidation are in part similar to those of retrieval. Indeed, some types of glutamate receptors and the activity of PKA, PKC and MAPKs are necessary for both consolidation and retrieval, and the modulation by D1 dopamine receptors, β-adrenoceptors, 5HT1_A_ receptors and cholinergic muscarinic receptors is similar in both cases^7,8,45,46^. But the nature and the timing of the role played by each signaling pathway in consolidation is different from that of retrieval^9^. In addition, other mechanisms crucial for encoding or consolidation of IA LTM like NMDA receptors and CaMKII are in general terms not necessary for memory retrieval.

Besides changes in the modulation of translation, brief inhibition of mTORC1 by rapa affects biological processes such as ion homeostasis, regulation of membrane potential, regulation of secretion and synaptic vesicle trafficking^47^. These findings suggest that acute inhibition of mTORC1 may greatly impact synaptic function and as a consequence impairs memory retrieval.

In conclusion, we found that the activity of mTORC1 close in time to memory recall is required for the normal expression of aversive and non-aversive LTM in the rat. Together with the seminal work of Nader group in amygdala^11^, our findings open new lines of research regarding the molecular mechanisms underlying memory retrieval.

## Materials and Methods

### Subjects

Male adult Wistar rats weighting 200–250 g on arrival (Facultad de Ciencias Exactas y Naturales, University of Buenos Aires) were housed in groups of five per cage and kept with water and food ad libitum under a 12 light/dark cycle (lights on at 7 A.M.) at a constant temperature of 23 °C. Experiments took place during the light phase of the cycle. Animals were handled for 3 min for 3 consecutive days before the experiment to avoid emotional stress.

All the experimental protocols used for this study followed the guidelines of the National Institutes of Health Guide for Care and Use of Laboratory Animals and were approved by the Animal Care and Use Committees of the University of Buenos Aires (CICUAL).

### Surgery

Rats were bilaterally implanted under deep ketamine/xylazine anesthesia (100 and 5 mg/kg, respectively) with a 22-gauge guide cannulae aimed to the dorsal CA1 region of the hippocampus (AP - 3.9 mm, LL ± 3.0 mm, DV - 3.0 mm from Bregma) or to the retrosplenial cortex (AP - 3.9 mm, LL ± 0.5 mm, DV - 1.8 mm from Bregma). Coordinates were based on Paxinos and Watson Atlas^48^. Cannulae were fixed to skull with dental acrylic. At the end of the surgery, animals were injected with a single dose of meloxicam (0.2 mg/kg) as analgesic and gentamicin (2.5 mg/kg) as antibiotic. Behavioral procedures took place 5–7 days after surgery.

### Inhibitory Avoidance Task

After recovery from surgery, IA was performed as described previously^22^. During training, rats were placed on a 5 cm high, 9 cm wide platform at left of a 47 × 25 × 30 cm opaque acrylic box whose floor was a grid made of a series of parallel 1 mm-caliber steel bars spaced 1 cm apart. As they stepped down onto the grid they received a 3 s, 0.7 mA scrambled foot shock and the latency to step down with all four paws was measured. Rats were tested for retention 2 h, 24 h, 2, 7, 14 or 28 days after training, depending on the experiment. In the TS the procedures were similar except that the foot-shock was omitted and the time spent on the platform was evaluated for a maximum of 300 s. Typical IA 24 h step-down latency was 150 ± 30 s. In the case where the reversibility of the rapa effect was examined, animals were retested 5 h after the first TS.

### Object Location Task

The OL task consisted of 3 days of 20 min habituation sessions in the absence of the objects, a 10 min TR and a 3 min TS. Before habituation, all rats were handled 2 min daily for 3 days. The experimental apparatus was a 60 cm wide × 40 cm long × 50 cm high acrylic box with a transparent frontal wall and hatched back wall, while laterals walls were white with different visual clues. On the TR day, the objects (two identical glass bottles) were located in the arena in two adjacent corners and animals were allowed to explore for 10 min. During TS, performed 24 h after training, one of the objects was switched to a new position and exploration time was recorded again. A rat was scored as exploring an object when it orientates its head towards the object with the nose within 1 cm of the object with behaviors including sniffing, touching and gnawing. Sitting on the object, looking up while resting against the object or any time where the rat simply propped the forepaws onto the object with the nose pointing away from it were not counted as exploration. Rats were excluded of the analysis if they explore one of the objects more than 65% of total objects-exploration time during TR. In both TR and TS, rats with total objects-exploration time lower than 10 s were excluded. The exploration time was measured using a hand stopwatch and results are expressed as a discrimination index: [Exploration time of new location (Tn) - Exploration time of familiar location (Tf)]/(Tn + Tf).

From rat to rat, the familiar or novel OL of the object in TS was counterbalanced. The box and the objects were thoroughly cleaned between trials.

### Open Field Test

To evaluate locomotor activity animals were exposed to an open field. The OF arena consisted in a square box of 50 × 50 × 39 cm, with black walls and floor, which was divided into nine quadrants by white stripes. Animals were left to explore for 5 min and the exploratory activity was measured as the number of crossings between squares and the number of rearings registered minute by minute.

### Drug Infusion

For intracerebral infusions, 30-gauge needles connected to Hamilton syringes were used. For rapa, the volume infused was 0.5 µl/side and the infusion rate was 0.5 µl/min. For eme, the volume infused was 1 µl/side and the infusion rate was 1 µl/min. Infusions were delivered through a needle extending 1 mm beyond the tip of the guide cannulae. Injectors were left in place for an additional min following infusion before they were removed carefully to minimize backflow. During the procedure the animals were slightly restrained with the hands, without provoking any evident stress. Drugs and doses were as follows: rapa (specific mTORC1 inhibitor) 60 nM/side and eme (irreversible protein synthesis inhibitor)^49^ 50 µg/side, both dissolved in saline. These doses were chosen based on previous findings^22,41^. Drugs were purchased from Sigma-Aldrich (USA).

### Cannula placement

Cannula placement was verified after the end of the behavioral procedures by infusions of 0.5 µl of a solution of 4% methylene blue in saline. Animals were killed by decapitation 15 min later and histological localization of cannula placement was performed, taking the extension of the dye as an indicator of the presumable diffusion of rapa. Infusions spread with a radius between 0.5 and 1.0 mm^3^ as described before^21,22,50^ was similar to that found using 3H-muscimol or fluorescent muscimol^51,52^ and is in agreement with published data from our group^53^, in which rhodamine labeled alpha-bungarotoxin infusions match those observed in the schema of Fig. 1a. Infusions were found to be correct (i.e., within 1.5 mm^3^ of the intended site) in 94% of the animals. Only the behavioral data from animals with the cannula located in the intended site were included in the final analysis.

### Data analyses

Statistical analysis and graphs of behavioral data were performed with unpaired Student’s t test when comparing two groups and one-way ANOVA Test followed by Newman-Keuls Comparison Test when comparing 3 or more groups using Graph Pad Prism 5® software (Graphpad, La Jolla, CA, USA). In all the cases, α level was set at 0.05. IA data in the bar graphs were expressed as mean ± SEM of training or test session step-down latency. In addition, training effect was always found significant; thus, for the sake of visual simplicity symbols showing significant differences between step-down latencies of training and testing were omitted.
